# Impact of the private sector on spatial accessibility to chest radiography services in Lima, Peru

**DOI:** 10.5588/ijtldopen.23.0460

**Published:** 2024-03-01

**Authors:** Y. Xiong, A.K. Millones, S. Farroñay, I. Torres, D. Acosta, D.R. Jordan, J. Jimenez, C. Wippel, H.E. Jenkins, L. Lecca, C.M. Yuen

**Affiliations:** ^1^ Division of Global Health Equity, Brigham and Women’s Hospital, Boston, MA, USA;; ^2^ Socios En Salud Sucursal Peru, Lima, Peru;; ^3^ Department of Global Health and Social Medicine, Harvard Medical School, Boston, MA,; ^4^ Department of Biostatistics, Boston University School of Public Health, Boston, MA, USA

Dear Editor,

Chest radiography (CXR) is an important tool for improving the diagnosis of TB in low- and middle-income countries (LMICs) with high rates of TB disease.^1^ However, in many such settings, public sector capacity for CXR is limited, and although additional radiography services exist in the private sector these may not be affordable to people with TB.^2,3^ Given the high use of private sector health services in many LMICs,^4^ private sector engagement is a key strategy for improving TB diagnosis and treatment.^5^ Engagement initiatives can encourage private facilities to provide free services through vouchers and contracting agreements with government programs.^6^ For example, in South Asia, private sector engagement has increased TB case detection.^7^ Also in Pakistan and Bangladesh, social enterprise TB screening centers offering low-cost CXR for people referred by private sector providers have detected TB disease among 15–20% of clients.^8,9^ However, the potential impact of private sector engagement on equality of access to services depends in part on the geographic location of private health facilities.

We have conducted an exploratory analysis to assess the extent to which engaging private health facilities to offer free CXR for TB diagnosis would impact the population of Carabayllo District of Lima, Peru. We obtained 2017 census tract maps and population counts from the Peru National Institute of Statistics and Informatics, Lima, Peru, along with a list of public health facilities with CXR services from the North Lima Regional Authority of the Ministry of Health. We identified private health facilities with CXR services from a survey of private health facilities in a previous study.^10^ We mapped the locations of these health facilities and estimated pedestrian travel distance following the road network from the geographic center of each census tract to each health facility using ArcGIS Pro v3.1.0 (ESRI, Redlands, CA, USA). We first assessed whether private facilities increased the percentage of the population with access to a facility with CXR using a χ^2^ test to compare the proportion of the population within a 3 km travel distance of a public facility to the proportion of the population within 3 km of any facility with CXR. We then used the enhanced two-step floating catchment area approach^11^ to calculate a spatial accessibility index to CXR that accounts for both distance from health facilities and the number of available health facilities per unit population. A Gaussian decay function was used to weight travel distances from census tracts to health facilities, applying weights of 1.00, 0.75, and 0.08 for distances of respectively 0–1 km, 1–2 km, and 2–3 km; distances over 3 km were not considered accessible given that people walk for transport in this urban area.^12^ We first calculated the accessibility index considering only public health facilities, then repeated the calculation taking into account both public and private facilities. We calculated the population-weighted median and interquartile range (IQR, the difference between population-weighted 25^th^ and 75^th^ percentiles) as measures of average accessibility and equality of accessibility.

**Figure.**
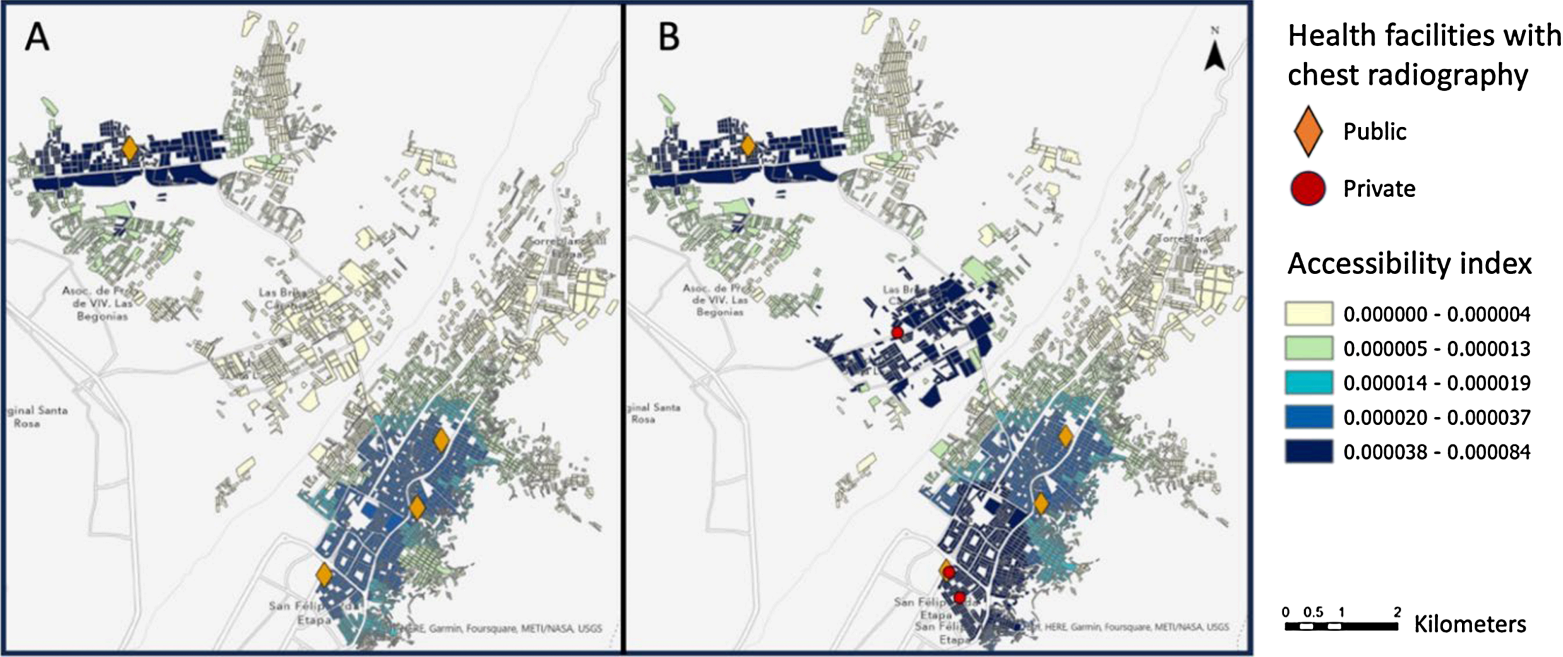
Spatial accessibility to chest radiography services in Carabayllo District, Peru. Census tracts with higher accessibility index values (darker colors) indicate greater access. In Panel **A)** only public health facilities are considered, whereas in Panel **B)** both public and private facilities are considered.

Using data for 270,454 residents of 3,253 census tracts within Carabayllo District, we found that 4 out of 12 public health facilities in the district had CXR services, and 3 private health facilities had CXR services. In total, 78% of the population was estimated to have at least one public health facility with CXR within a 3 km travel distance, whereas 86% had at least one health facility of any type with CXR within a 3 km travel distance (*P* < 0.001). Figure A shows the tract-level accessibility index for public health facilities with CXR services; Figure B shows the accessibility index for both public and private facilities. One of the private facilities was located in an area where the local public health facility lacked radiography services, so it would benefit the local population if the private services were more affordable. The two other private facilities were located in close proximity to public radiography services, increasing access for census tracts that already had relatively high accessibility indices. With only public facilities, the population-weighted median accessibility index value was 11.4 x 10^-6^ (IQR 21.2 x 10^-6^ ). With all facilities, the population-weighted median accessibility index value was 18.9 x 10^-6^ (IQR 43.4 x 10^-6^). Thus, although average accessibility increased, the doubling of the IQR shows increased inequality of access.

Given that only geographic accessibility and not other dimensions of healthcare access (such as convenience or acceptability of private vs. public services) were considered in this study, this simple exploratory analysis has limitations in estimating true access to CXR services. We also did not take into account the possibility of vehicular transport to health facilities, alternative distance cutoffs or impedance weights, or regulations around which public facilities people use. However, we believe that the conclusions drawn from comparing the two scenarios are valid as the same simplifying assumptions were made to calculate accessibility in both.

In conclusion, our analysis shows how considering spatial accessibility to services can help to inform interventions to improve access to CXR services. Although engaging private facilities to offer free CXR services in Carabayllo District could increase overall access to CXR, this risks exacerbating disparities in spatial accessibility. Work in other countries has shown that engaging the private sector can be effective in improving access to services,^6^ but that broad private sector engagement programs may worsen existing disparities.^13^ Moreover, there are areas where CXR services are not available in either the public or private sector. In these ‘radiography deserts’, complementary approaches such as mobile units may ensure access to CXR.^14,15^ Thus, to improve overall access to CXR and the equality of access, program planners should begin with an analysis of spatial accessibility and engage private providers in areas underserved by the public sector. This will also help to understand if it is necessary to deploy mobile diagnostic units to areas lacking both public and private sector radiography services.
